# Controllable Production of Natural Silk Nanofibrils for Reinforcing Silk-Based Orthopedic Screws

**DOI:** 10.3390/polym15071645

**Published:** 2023-03-25

**Authors:** Shuqin Yan, Li He, Abdul Moqeet Hai, Zhanao Hu, Renchuan You, Qiang Zhang, David L. Kaplan

**Affiliations:** 1 State Key Laboratory of New Textile Materials and Advanced Processing Technologies, School of Textile Science and Engineering, Wuhan Textile University, Wuhan 430200, China; 2 Institute of Polymer and Textile Engineering, Quaid-e-Azam Campus, University of the Punjab, Lahore 54590, Pakistan; 3 Department of Biomedical Engineering, Tufts University, Medford, MA 02155, USA

**Keywords:** natural silk nanofibrils, mechanical properties, screws, bionanocomposites

## Abstract

As a natural high-performance material with a unique hierarchical structure, silk is endowed with superior mechanical properties. However, the current approaches towards producing regenerated silk fibroin (SF) for the preparation of biomedical devices fail to fully exploit the mechanical potential of native silk materials. In this study, using a top-down approach, we exfoliated natural silk fibers into silk nanofibrils (SNFs), through the disintegration of interfibrillar binding forces. The as-prepared SNFs were employed to reinforce the regenerated SF solution to fabricate orthopedic screws with outstanding mechanical properties (compression modulus > 1.1 GPa in a hydrated state). Remarkably, these screws exhibited tunable biodegradation and high cytocompatibility. After 28 days of degradation in protease XIV solution, the weight loss of the screw was ~20% of the original weight. The screws offered a favorable microenvironment to human bone marrow mesenchymal stem cell growth and spread as determined by live/dead staining, F-action staining, and Alamar blue staining. The synergy between native structural components (SNFs) and regenerated SF solutions to form bionanocomposites provides a promising design strategy for the fabrication of biomedical devices with improved performance.

## 1. Introduction

Titanium alloy, stainless steel, and ceramics are the current state-of-the-art materials for bone fixation due to their excellent mechanical properties and implantability. However, their use involves certain complexities such as limited osseointegration, stress shielding, inflammatory response, and in some cases need for a second surgery for implant removal [1]. In addition to that metals are corrosive causing inflammation and pain; they are temperature sensitive causing discomfort; and in pediatric patients, the metal implants may migrate resulting in inflammation in healthy parts of the body [2]. Recently, the use of resorbable bone plates and screws composed of poly(L-lactic acid) and poly(glycolic acid) have garnered interest as they obviate the need for implant removal and result in improved bone remodeling [3,4]. However, their acid degradation products usually are regarded to create osteolysis, cyst, and incomplete bone reconstruction [5,6]. These bone fixation system failures underscore the need for bioinspired materials and strategies to enhance the osseointegration of implants and speedy bone regeneration.

Silk fibroin (*Bombyx mori* silk) has emerged as a promising biomaterial for the regeneration of load bearing tissues such as cartilage, ligament, and bone on account of its biocompatibility, robust mechanical properties, and controllable degradation rates [7]. Particularly, in terms of bone tissue engineering, SF materials have shown accelerated bone regeneration, increased tissue vascularization in in vitro models of bones, and enhanced osteogenic differentiation of human mesenchymal stem cells [8]. As a structural protein material, SF offers the unique advantage of superior mechanical properties (tensile strength 0.3–1.3 GPa and elongation at the break 4–38%) that stem from a mesoscopic hierarchical assembly of β-crystallite networks or nanofibrils [9,10]. It follows that nanofibrils are key structural elements that determine the macroscopic performance of SF materials.

Molecular dynamics (MD) simulations have become an increasingly popular tool for investigating the mechanical properties of biomolecules, including silk fibroin [11]. Silk fibroin is a fibrous protein that is the major structural component of silk, which is produced by various species of spiders and insects. Silk fibroin has unique mechanical properties, including high tensile strength, elasticity, and toughness, making it a promising material for various applications, such as in textiles, biomedical engineering, and nanotechnology. Several studies have used MD simulations to investigate the mechanical properties of silk fibroin, including its response to deformation, fracture, and stress relaxation [12,13,14]. These simulations have provided valuable information on the structural and mechanical behavior of silk fibroin at different length scales, from the molecular to the macroscopic level [14]. This progress encourages the design of homogeneous reinforced silk composites based on building blocks and the hierarchical structure of the silks.

Generally, SF fibers are converted into their regenerated solution form that can subsequently be reconstituted to construct macroporous 2D or 3D architectures for further use in biomedical applications. Although the main components in regenerated SF materials are the same, their mechanical performance is inferior to their natural counterparts due to the unwanted degradation of native protein structure during the dissolution process [15]. In this context, several attempts have been made to isolate SNFs from natural silk fiber while preserving their sophisticated hierarchical architecture. Strenuous conditions and agents such as HFIP [16], CaCl_2_-formic acid [17], NaOH-urea [18], NaClO [19], and urea-guanidine hydrochloride systems [20] have been used to isolate SNFs. Although these approaches have been successful, the methods tend to be tedious and difficult to scale up. Importantly, a relatively lower length-to-diameter ratio of SNFs is a matter of concern with the existing methods [21]. Therefore, the development of a system that could effectively swell and disintegrate SNFs without losing their characteristic ultra-structure and thus mechanical properties could provide a significant advance in designing new silk-based materials for load bearing applications.

In this study, we hypothesized that natural SNFs can be used as a reinforcement to improve the mechanical properties of pure silk nanocomposites. As described in our previous work [22], the SNFs were exfoliated using a ternary solution system [23] that weakened the binding forces between SNFs. On the other hand, a regenerated SF solution was prepared by dissolving silk in a Ca^2+^-formic acid solvent [24]. Finally, the silk bionanocomposite screws were prepared by reinforcing the regenerated SF solution with the SNFs. These devices presented surprising compression modulus and cytocompatibility as determined by mechanical properties tests and cell culture in vitro, respectively.

## 2. Materials and Methods

### 2.1. Exfoliation of Natural Silk Nanofibers (SNFs) 

**Figure 1:**
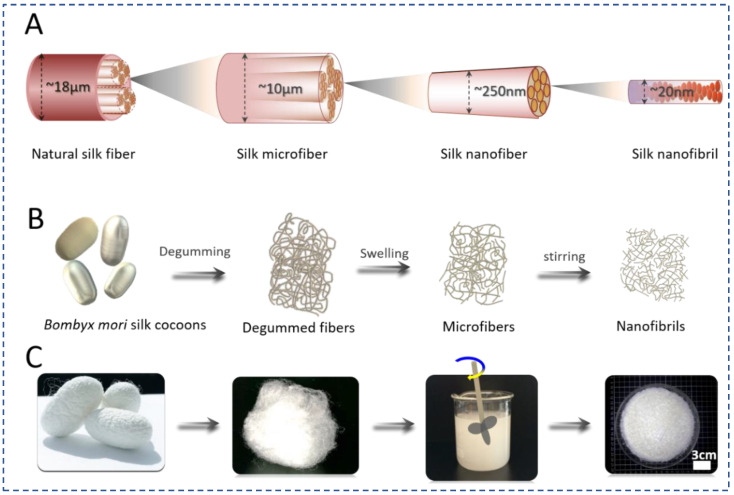
Schematic illustration of the SNFs fabrication process. (**A**) Schematic showing the fabrication of SNFs, (**B**) The process of SNFs via mechanical shearing, (**C**) the morphology changes of the silk fibers during SNF preparation from silk fibers and microfibers.

*Bombyx mori* silk cocoons (Huzhou, Zhejiang, China) were boiled three times in 0.06% (*w*/*v*) Na_2_CO_3_ solution for 30 min to remove sericin. The degummed silk fibers were washed with distilled water thoroughly and dried at 60 °C. The natural SNFs were prepared by an established procedure [25] as shown in Figure 1. Briefly, the dried degummed silk fiber was treated by Ca(NO_3_)_2_:CH_3_CH_2_OH: H_2_O (0.25 M Ca(NO_3_)_2_; molar ratio, 1:2:8) blend solution at 45 °C for 6 h. The swollen silks were mechanically disintegrated to obtain SNF suspension. After thoroughly washing and drying, the natural SNFs were obtained for further characterization and use.

### 2.2. Preparation of Silk Screws 

As described in our previous work [25], the degummed SF fibers were directly dissolved into 4.5% CaCl_2_-formic acid (Ca^2+^-FA) solution to obtain the 20% (*w*/*v*) SF Ca^2+^-FA solution at room temperature. Natural SNFs dispersed in 0.5 mL water (to avoid rapid dissolution of SNF in SF Ca^2+^-FA solution) were mixed with 20% SF Ca^2+^-FA solution to obtain a final SNF to SF solution ratio of 5%, 10%, 20%, and 40% (*w*/*w*). To homogenize, the mixture was vortexed for 10 min. The mixture was placed into dialysis tubes (molecular cut off: 3500 Da, diameter of 15 mm, Viskase, Lombard, IL, USA) and dialyzed for 24 h to form hydrogel nanocomposites based on our previous procedure [24]. The hydrogels were dried in an oven at 60 °C overnight to obtain silk rods. The silk rods were polished and processed into silk screws by CNC lathes (Variable Speed Lathe WM210V, Germany) for further characterization. The silk screws with 5%, 10%, 20%, and 40% SNFs was represented by the 5%@SNF, 10%@SNF, 20%@SNF, and 40%@SNF, respectively.

### 2.3. Morphology and Mechanical Properties of SNFs, Silk Screws 

To observe the morphology of the SNFs and silk screws, digital camera photos and scanning electron microscopy (SEM, Zeiss, Germany) were employed to observe cross-sections and surfaces after sputter coating for 100 s. The atomic force microscope (AFM, SPM9700, Shimadzu, Japan) was used to characterize the diameter of SNFs. The scan area was 5 μm × 5 μm. The compressive stress and modulus of the silk rods (r = 3 mm, height = 7 mm) were performed using a tensile testing machine (Instron 3366, Norwood, MA, USA) with a 10 kN capacity load cell. Prior to testing, all samples were hydrated by submerging them in PBS solution at 37 °C for more than 72 h. The diameter of the silk rods was measured using a micrometer before and after immersion in PBS. The maximum stress was 7 kN at a rate of 5.0 mm min^−1^. 

### 2.4. Biodegradation of the Silk Screws In Vitro

Protease XIV (EC 3.4.24.31, Sigma-Aldrich, Shanghai, China) was used for biodegradation studies as it has been shown to be effective in the digestion of casein as compared to trypsin, chymotrypsin and several other proteases [26]. Silk rods were incubated in a shaker at 37 °C in a solution of protease XIV (20 ± 1 mg, pH = 7.4, 3.5 U/mg) and PBS for 28 days. Each specimen contains an approximately equivalent dry mass (1.00 ± 0.02 g) of silk-based rods and each specimen has five replicates. During the degradation, the enzymatic aqueous solutions were replenished weekly. The samples were harvested at different time points followed by rinsing with distilled water 3 times. The residual mass was dried at 60 °C for 24 h to determine the degradation ratio of silk rods.

### 2.5. Structure and Stability Analysis of Silk Screws

To investigate the structural change of the SF nanocomposites, the mixture of SNFs and SF was cast into films by solvent evaporation as a control group. The films were treated with deionized water as an experimental group. Fourier transform infrared spectroscopy (FTIR) in the 4000–400 cm^−1^ wavenumber range was collected with a NicoletAvatar-IR360 (Thermo Nicolet Corporation, Waltham, MA, USA) with 128 scans. Additionally, Raman spectroscopy was also performed to determine the molecular orientation in polymer fibers. The composite films were cut into micro-particles with a radius of less than 40 μm and samples were fixed on a machine holder. Spectra were recorded on single SF fibers via a Dilor LabRam-1B Raman microscope (Bruker, Germany) using a 632.81 nm excitation laser beam of He-Ne laser with an energy of 3 mW. The scanning time was 200 s for each sample. 

The dried silk rods reinforced by SNF were milled into a powder using a mincer. Differential scanning calorimetry (DSC), thermogravimetry (TG), and differential thermal analysis (DTA) of the rods were measured by Simultaneous Thermal Analyzer (204F1, Netzsch, Germany). The samples were heated from 30 °C to 400 °C at a heating rate of 10 °C/min in an inert gas N_2_ environment. 

### 2.6. Cell Culture

Commercial human bone mesenchymal stem cells (hBMSCs) isolated from fresh bone marrow aspirate (Lonza, NJ, USA) of a healthy, nonsmoking, young male was used [27]. The culture medium contained Dulbecco’s modified eagle medium (DMEM) supplemented with 10% fetal bovine serum (FBS), 1% non-essential amino acids, 1% antibiotic/antimycotic (100 U mL^−1^ penicillin, 100 mg mL^−1^ streptomycin, 0.25 mg mL^−1^ fungizone) and 1 ng mL^−1^ fibroblast growth factor-basic. For cell seeding, the silk screws were blotted after immersion in PBS for 24 h followed by autoclaving for sterilization. Cells at passages 3 were seeded at 1 million per silk screw (50 μL volume) and allowed to attach for 2 h prior to flooding the composites with growth media. The growth medium contained DMEM supplemented with 10% FBS, 1% non-essential amino acids, and 1% antibiotic/antimycotic. All cell cultures were incubated at 37 °C with 5% CO_2_. The medium was replenished every 2.5 days. No cell-loaded silk screws served as controls. Following the manufacturer’s protocol, hMSC proliferation was monitored by Alamar blue dye reduction assay. Viable cells in the composites after 9-day culture were observed using a live/dead assay kit. Actin expression of hMSCs in 3D cell culture was visualized by fluorescence F-actin staining under laser scanning confocal microscopy (LSCM, Nikon A1r, Tokyo, Japan). 

### 2.7. Statistical Analysis

Data were presented as means ± standard deviation (SD) for all experiments. To determine the statistical significance of the analysis, an ANOVA (*t*-test) was performed and the results were considered statistically significant for a *p*-value < 0.05.

## 3. Results and Discussion

### 3.1. Exfoliation of SNFs

**Figure 2:**
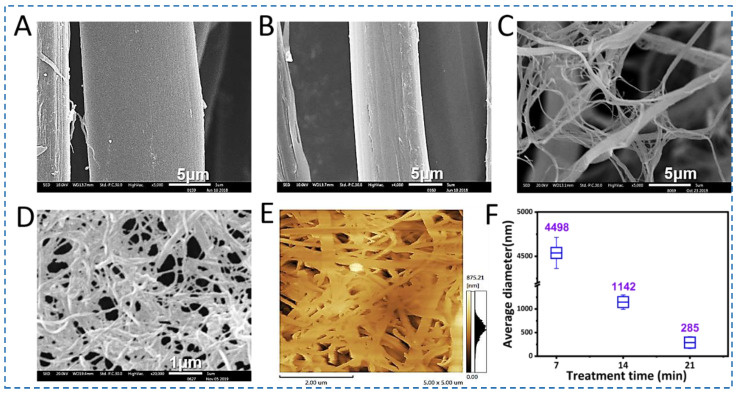
The morphology and diameters of SNFs and SNF films. (**A**–**D**) represent SEM images of SNFs film with different magnifications, (**E**) represents AFM images of SNFs film, and (**F**) the average diameters of the SNFs treated with different treatment times. Scale bars for (**A**–**E**) are 5 μm, 5 μm, 5 μm, 1 μm, and 2 μm, respectively.

As a unique fibrous protein biopolymer with a hierarchical structure, SF fibers are assembled by various binding forces, including hydrogen bonds [28,29], hydrophobic interactions [30], ionic bonds [31], and Van der Waals’ forces [32]. Owing to the presence of these binding forces at all levels of structural hierarchy, it is a significant challenge to controllably reverse engineer SF fibers to exfoliate SNFs while keeping their structure undamaged. A ternary solvent consisting of Ca(NO_3_)_2_:CH_3_CH_2_OH: H_2_O (0.25 M Ca(NO_3_)_2_; molar ratio, 1:2:8) was employed to weaken the binding forces followed by appropriate mechanical shearing to generate the SNFs [25]. The degummed silk fibers were treated with this ternary solution at 45 °C for 6h to swell and form microgaps between SNFs, which facilitated ethanol access during mechanical shearing [33]. Once the silk fibers were swollen, Ca^2+^ carried additional water molecules into the silk semi-crystalline and crystalline regions to further weaken binding forces between SNFs [17,34]. Finally, the silk fibers were disintegrated into SNFs after mechanical shear. As shown in Figure 2, after the treatment with the ternary solvent, the diameter of the silk fibers decreased significantly due to the exfoliation of nanofibers from the surface of the fibers (Figure 2A,B). The surface of the treated fibers showed rougher with a few small pitting, followed by deconstruction into micro- and nano-scale fibers (Figure 2C–E).

To observe the morphology of the SNFs, the SNFs suspension was converted into a film by vacuum-filtration (200 mL, 0.2 wt%). The film demonstrated homogeneous micro- or nano-fiber networks (Figure S1). It was also observed that owing to 20 min shearing, some of the silk fibers dissolved, resulting in silk fibroin solution coating of the films (Figure S1L). However, the nanofibers showed more uniform features and were distinct at 15 min treatments (Figure S1D). The exfoliated silk nanofibers showed a diameter distribution in the range of 285 nm as determined by the AFM image (Figure 2E). Subsequently, these SNFs prepared by 15 min shearing were used as reinforcing material for silk screw fabrication. 

### 3.2. Preparation and Morphology of Silk Screws of Silk Screws

**Figure 3:**
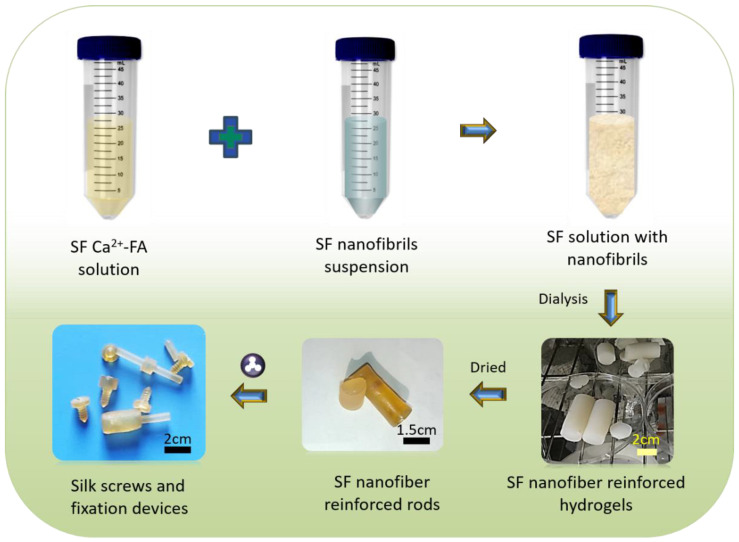
Schematic illustration of the silk screw fabrication process. The process could be divided into six steps. It is simple and easy-handle. The degummed silk fibers were directly dissolved intoCa^2+^-FA solution. The natural SNFs were mixed with the silk Ca^2+^-FA solution. The mixture was placed into dialysis tubes and dialyzed to form silk hydrogels. The hydrogels were dried in an oven to obtain silk rods. After milling, the silk rods were processed into silk screws.

Silk blocks with high density have previously been milled into silk screws, pins, and plates for hard tissue repairs [35,36]. However, this was carried out using regenerated silk fibroin solutions, requiring time-consuming concentration steps. Herein, the silk fibroin fibers were directly dissolved into Ca^2+^-FA solution to obtain silk fibroin solution with high concentration. To fabricate silk screws, SNFs were added to the silk fibroin solution (Ca^2+^-FA system) to obtain silk-based nanocomposites. These silk nanocomposites were cast in a mold to form silk rods which can later be machined into various types of orthopedic devices such as pins, and screws (Figure 3). Silk screws (10%@SNF) were machinable and their surface was also threaded as shown in Figure 3. 

**Figure 4:**
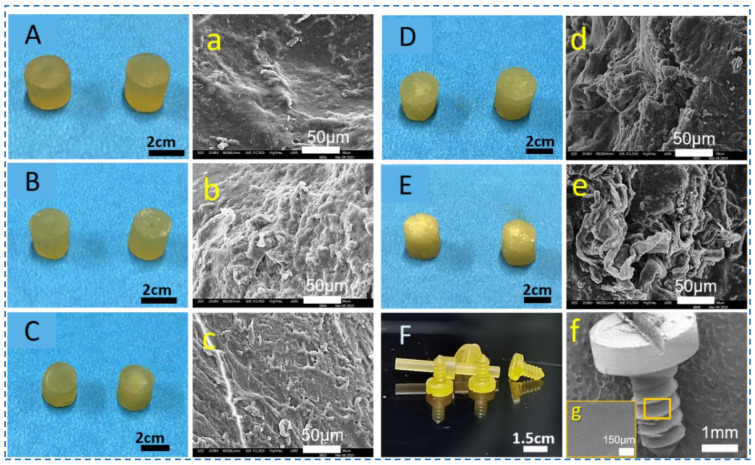
The cross sections of the silk rods and screws with different SNFs content. (**A**,**a**) silk rod without SNFs, (**B**,**b**) silk rod@5 wt% SNFs, (**C**,**c**) silk rod@10 wt% SNFs, (**D**,**d**) silk rod@20 wt% SNFs, (**E**,**e**) silk rod@40 wt% SNFs, and (**F**,**f**) SEM images of the silk screws.

The increase in SNF percentage decreased the transparency of silk nanocomposite. When the SNF content was 40%, the cross-section of the silk rod has a granular texture caused by crumbling SNF grains at the edges of the silk rods (Figure 4E). Additionally, the silk rod showed brittleness and a low density (Figure 4E). The SEM images also exhibited a rough surface at the cross-section of silk rods reinforced by SNFs were observed, while silk rods without SNFs were found to be relatively smooth (Figure 4A–e). As shown in Figure 4F, the silk screw was successfully machined from silk rods. These silk screws (major diameter ~1.5 mm) remained intact during the machining process and the screws had precise threads with sharp edges (Figure 4F). The surfaces of screws on threads and grooves appeared uniform. The general appearance of the screws displayed a relatively homogeneous appearance, while a rougher surface was on fine features (Figure 4F). These features contribute to early fixation and long-term stability in defect sites of bone due to the mechanical interlocking between the screws and bone ingrowth [37]. Meanwhile, these rough topological structures also offer a favorable surface for cell attachment and biological response to silk screws [38]. 

### 3.3. Mechanical Performance and Degradation of Silk Screws 

**Figure 5:**
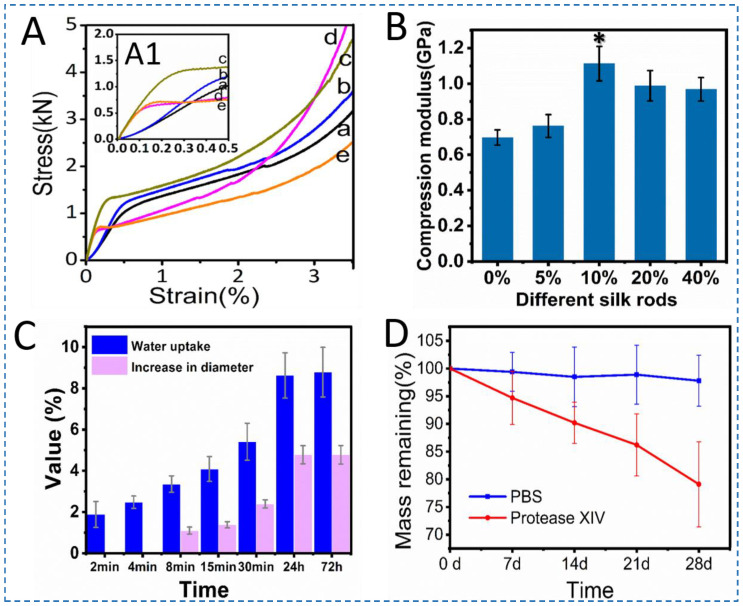
Mechanical and biodegradable properties of different silk rods. (**A**,**A1**) represent stress-strain profiles of different silk rods ((a–e) indicate silk rods with different SNFs at 0%, 5%, 10%, 20%, and 40%, respectively), (**B**) represents compression modulus of the silk rods(* *p* < 0.05), (**C**) shows the diameter increase of the silk rod @10% SNFs immersed into PBS for different time (pH = 7.4), (**D**) shows biodegradation of the silk rod @10% SNFs in protease XIV solution and PBS for 28 days.

For fixation in bone tissue engineering, mechanical properties are critical in terms of the clinical utility of an implant. A remarkable feature of these nanocomposite silk rods is their machinability. To evaluate mechanical performance, the silk rods having a radius and height of 3 mm and 7 mm were selected. Compression tests in the hydrated state showed a significant nonlinear relationship in the stress-strain curves, indicating that the ability to resist deformation varied with SNF content (Figure 5A,B, Table 1). Considering the highest compression modulus of silk rods with 10% SNFs (*p* < 0.05), the stiffness of rods improved significantly by the integration of the SNFs (1.1 GPa). The presence of SNFs served as fillers thereby increasing the stiffness and also contributing to preventing stress concentration for the silk bionanocomposites. The diameter increase and water uptake of the silk rods were 4.1% and 8.7% respectively after 72 h soaking in PBS at pH 7.4 (Figure 5C). These results suggest that silk rod 10%@SNFs possesses high stability under physiological conditions, which are beneficial in terms of their utility as medical devices in vivo. 

Considering the unique mechanical properties of the SNF-reinforced biocomposites, it is worth noting that these MD simulations of silk fibroin have been conducted using a variety of methods and techniques, each with its own strengths and limitations. One approach commonly used in MD simulations of silk fibroin is the use of force fields [11], which are mathematical models that describe the interactions between atoms and molecules in the protein. These force fields can be parameterized to reproduce experimental data on the properties of silk fibroin, such as its elastic modulus, tensile strength, and fracture behavior. Another technique used in MD simulations of silk fibroin is steered molecular dynamics, which involves applying a force to a specific region of the protein and monitoring its response to deformation. This technique has been used to study the unfolding and refolding of silk fibroin molecules, as well as their response to tensile and compressive loads [12,13,14]. By comparing the results of these simulations with experimental data, researchers can validate their models and refine their understanding of the mechanical behavior of silk fibroin. In brief, MD simulations have provided valuable insights into the mechanical properties of silk fibroin, and their use is likely to continue to advance our understanding of this fascinating protein and its potential applications.

As a bone repair device, both screw shear and torsional breaking can have serious consequences for the functionality of a system and its overall safety, so it is important to design and maintain components to prevent these types of failures from occurring. In this study, based on the principle of homogeneous reinforcement, we use natural nanofibers to enhance the mechanical properties of the screws by reinforcing regenerated silk fibroin materials. Studies have shown that the silk screw has a high resistance to shear breaking and torsional breaking [27,36]. The remarkable breaking behaviors of the silk screw are due to its unique structure and composition. Although, the shear and torsional breaking behavior of the silk screws are not shown in this study. Considering that the silk screw is composed of a bundle of nanofibers that may be wrapped around each other. This structure is supposed to provide the silk screw with both strength and flexibility, allowing it to resist shear and torsional forces. In the future, we will further characterize these properties carefully.

The degradation behavior of biomaterial is critical to the success of tissue regeneration. Protease XIV was selected as the model enzyme due to its powerful digestion of casein of silk fibroin. It is worth mentioning that the concentration of the enzyme (1.0 UmL^−1^) used is higher than the human body level [39]. Silk rod @10wt% SNFs biodegraded in protease XIV and PBS solution after 28 days. The silk rods were found more resistant to degradation in PBS as compared to the protease XIV solution. The weight loss followed a clear trend after exposure to protease XIV solution. After 28 days of biodegradation, the silk rods retained ~80% (*w*/*w*) of the original mass (Figure 5E). In PBS, the weight loss of the sample gradually increased from 0 to 2.5% due to swelling effects and shearing by the shaker at 37 °C (Figure 5E). Considering the use of silk screws as fixation systems, high degradation resistance is important to the screw performance as an implanted orthopedic device in bone tissue engineering.

### 3.4. Structural Analysis of Silk Rods

**Figure 6:**
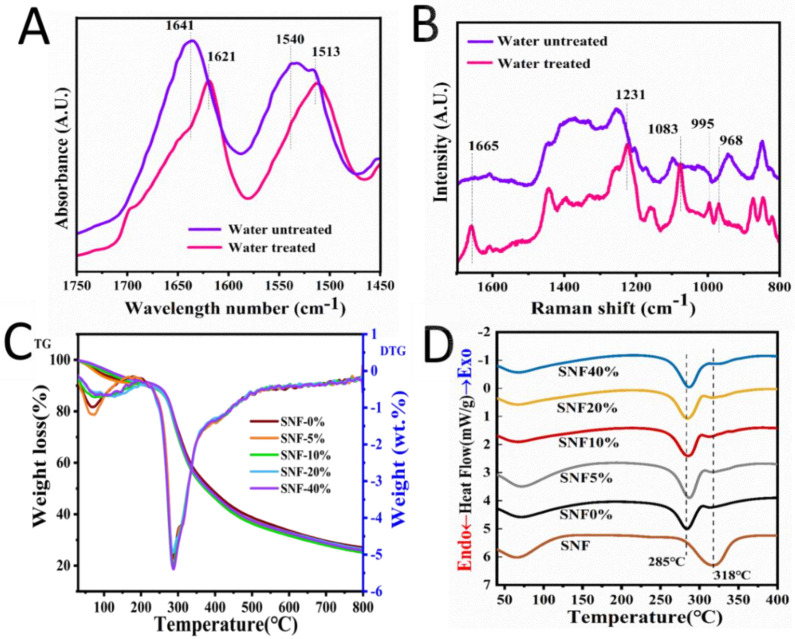
Structure analysis and thermal stability of the silk rod derived from the Ca^2+^-FA system. (**A**) represents ATR-FTIR and (**B**) Raman spectra of the silk hydrogel derived from Ca^2+^-FA system treated and untreated by water (**C**), represents TG-DTG of dried silk rod with different SNF, and (**D**) represents DSC of dried silk rod with different SNF.

To characterize the structural changes during the process of making screws, the mixture of the SNFs and SF Ca^2+^-FA solution was cast into films via solvent evaporation as a control group. The films were treated with deionized water as an experimental group. As shown in Figure 6A, the absorption bands of the treated film shifted to lower wavenumbers. After water treatment, the spectra of the film absorption band near 1641 cm^−1^ gradually shifted to 1621 cm^−1^, while absorption bands near 1540 cm^−1^ shifted to 1513 cm^−1^ (Figure 6A). These changes showed that random coils and α-helix conformations transformed into β-sheets after water treatment. The natural silk fiber is insoluble in water, while it can be dissolved in strong acid (pH~2) for instance Ca^2+^-FA solution [24]. When the SF Ca^2+^-FA solution was immersed in water, solvent exchange triggered the SF conformation transition to form insoluble hydrogel by inducing macromolecule folding and crystallization [24]. This change was also verified by Raman spectra (Figure 6B). After water treatment, new spectra were observed around 1665 cm^−1^, 1231 cm^−1^, 1083 cm^−1^, 995 cm^−1^, and 968 cm^−1^ which pointed to the presence of β-sheet structures [40]. Increase of β-sheet content after water treatment was confirmed due to a conformation transition within the silk rods.

It can be seen from the thermogravimetric curve in Figure 6C,D. The weight loss at about 100 °C was mainly caused by the evaporation of free water; The weight loss peak of the homogenous rod was relatively large from about 285 °C, which was due to the sharp decline in the quality of protein due to thermal decomposition. The weight loss peak of pure SNF was about 318 °C, and that of SNF/SF rod is about 285 °C, which is lower than that of pure SNF. With the introduction of SNF, its endothermic decomposition peak shifts back, indicating that SNF can improve the thermal stability of SNF/SF three-dimensional rods. Due to both the reinforcement and the base material being derived from silk fibers, contributes to establishing a solid and stable interface with high compatibility.

### 3.5. Cytocompatibility of the Silk Screws

**Figure 7:**
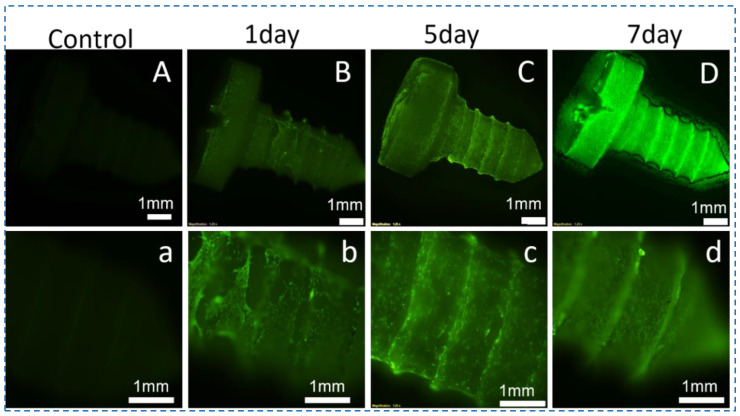
The stereoscopic fluorescence microscope images of hBMSCs growth morphology on the silk screws @10 wt% SNFs at different time points. (**A**,**a**) represent the silk screw unloaded cells. The cells were stained by a live/dead assay kit. Live/dead staining of hBMSCs cultured on silk screws in growth medium at day 1 (**B**,**b**), day 5 (**C**,**c**), and day 7 (**D**,**d**). Strong green fluorescence without visible red fluorescence demonstrated the cytocompatibility of the silk screws.

**Figure 8:**
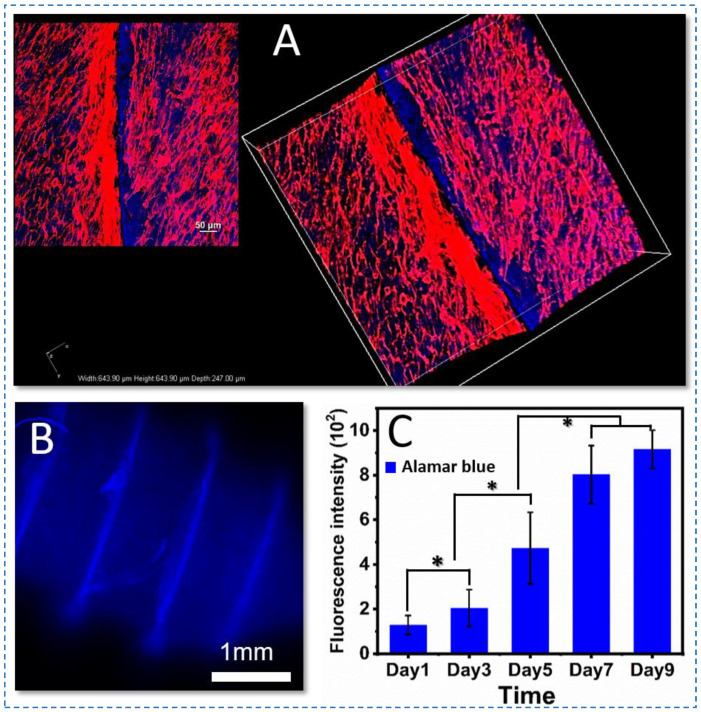
Fluorescence staining of actin (red) and nuclei (blue) of hBMSCs on the silk screws composites in growth medium at day 7 (**A**). (**B**) represents silk screws unloaded hBMSCs. The cells spread out actin filaments, evenly distributed on the screws after 7-day culture. Alamar blue assay (**C**) showed the metabolic activity and proliferation of hBMSCs on the screws (n > 3) over a 9-day culture period. Silk screw unloaded cells served as a control (* *p* < 0.01).

Stem cell transplantation together with tissue engineering is increasingly becoming a promising treatment strategy. However, direct transplantation of stem cells without scaffolds has yielded poor clinical outcomes [41]. Stem cells and biomaterials were combined to allow cell attachment onto substrates and the production of constructs with similar functional and morphological characteristics to natural tissue. The intricate relationship between silk screws and hBMSCs in vitro must be examined during the bone repair process to support successful bone formation. Herein, hBMSCs seeded on the silk screws@10% SNFs for 9 days were used to assess cytocompatibility (Figure 7 and Figure 8). On day 1, the cells attachment were found to be uniform and compatible. There were no dead cells observed by using a live/dead assay kit (Figure 7). After 7 days of culture, the cells exhibited the confluent culture on the screw surface (Figure 7). After 9 days in culture, the hBMSCs exhibited favorable metabolic activity and spread and aligned with the grooves of the screws with a uniform distribution (Figure 8A). The F-actin staining also revealed cellular attachment and cytoskeletal spreading on the surface of the silk screws. While no red fluorescence light was observed in silk screws (Figure 8A,B). Alamar blue assay showed the proliferation of hBMSCs on the silk screws during culture (Figure 8C). A significant increase in fluorescence intensity from days 3 to 7 was observed, indicating cell favorable metabolic activity and proliferation (*p* < 0.01). These results suggest that the screws support the hBMSCs growth and metabolism. In this study, the ternary solvent swelling and mechanical shearing were employed to generate natural SNFs. The ternary solvent system utilized in the present study was recyclable and biocompatible in SF scaffolds for tissue engineering.

Furthermore, the gross appearances of the screws showed relatively uniform morphology, while a rough surface was on high magnification (Figure 4g). These features are beneficial to cell attachment and spread ((Figure 8A). In brief, the silk biocomposite screws with high mechanical properties, biodegradability, and cytocompatibility were prepared by SNF reinforcement, offering potential applications in biomedical devices.

## 4. Conclusions

In this study, natural silk nanofibers produced by a facile strategy were employed as reinforcement for SF solution to prepare pure silk nanocomposite for the fabrication of silk screws. Natural SNFs were exfoliated by using a ternary solvent treatment followed by mechanical shearing. The as-prepared nanocomposite silk screws offered superior mechanical properties and it was observed that an increase in SNF content in nanocomposite increased the compression modulus of silk screws. The structural analysis of nanocomposite films revealed the presence of β-sheet conformations. The in vitro degradation studies established the degradation profile of silk screws in protease XIV. The cytocompatibility studies exhibited hBMSCs attachment and proliferation on silk screws. These SNF-based nanocomposites with enhanced mechanical performance can potentially be used in load bearing tissue regeneration applications such as screws for orthopedic fixation.

## Figures and Tables

**Table 1:** Mechanical properties of different silk rods.

SAMPLES	SNF0%	SNF5%	SNF10%	SNF20%	SNF40%
Stress (kN)	1.02	1.20	1.37	0.79	0.75
Compression modulus (Gpa)	0.697 ± 0.043	0.762 ± 0.064	1.112 ± 0.097	0.988 ± 0.085	0.968 ± 0.066

## Data Availability

The data that support the findings of this study are available from the corresponding author upon reasonable request.

## Supplementary Materials

The following supporting information can be downloaded at: https://www.mdpi.com/article/10.3390/polym15071645/s1, Figure S1: The SEM images of silk micro-nanofibers and SNF films. (A–D) represents the gross appearance of the SNFs obtained by different treatment times, (E–H) Silk fibers treated for different times at 30 °C. (I–L) Silk fibers treated for different times at 35 °C, scale bar = 2 μm.
