# Supplementary material for: Controllable Production of Natural Silk Nanofibrils for Reinforcing Silk-Based Orthopedic Screws
Source: Polymers (Basel). 2023 Mar 25;15(7):1645. doi: 10.3390/polym15071645 (PMC10096991; doi:10.3390/polym15071645)
Supplement: Supplementary file 1 [file polymers-15-01645-s001.zip › polymers-2256921-supplementary.pdf]

Supporting Information

Controllable Production of Natural Silk Nanofibrils for Reinforcing Silk-Based Orthopedic Screws

Shuqin Yan [1,†], Li He [1,†], Abdul Moqeet Hai [2], Zhanao Hu [1], Renchuan You [1], Qiang Zhang [1,*] and David L. Kaplan [3]

[1] State Key Laboratory of New Textile Materials and Advanced Processing Technologies, School of Textile Science and Engineering, Wuhan Textile University, Wuhan 430200, China
[2] Institute of Polymer and Textile Engineering, Quaid-e-Azam Campus, University of the Punjab, Lahore 54590, Pakistan
[3] Department of Biomedical Engineering, Tufts University, Medford, MA 02155, USA
* Correspondence: author: zhangq12041008@163.com
† These authors contributed equally to this work.

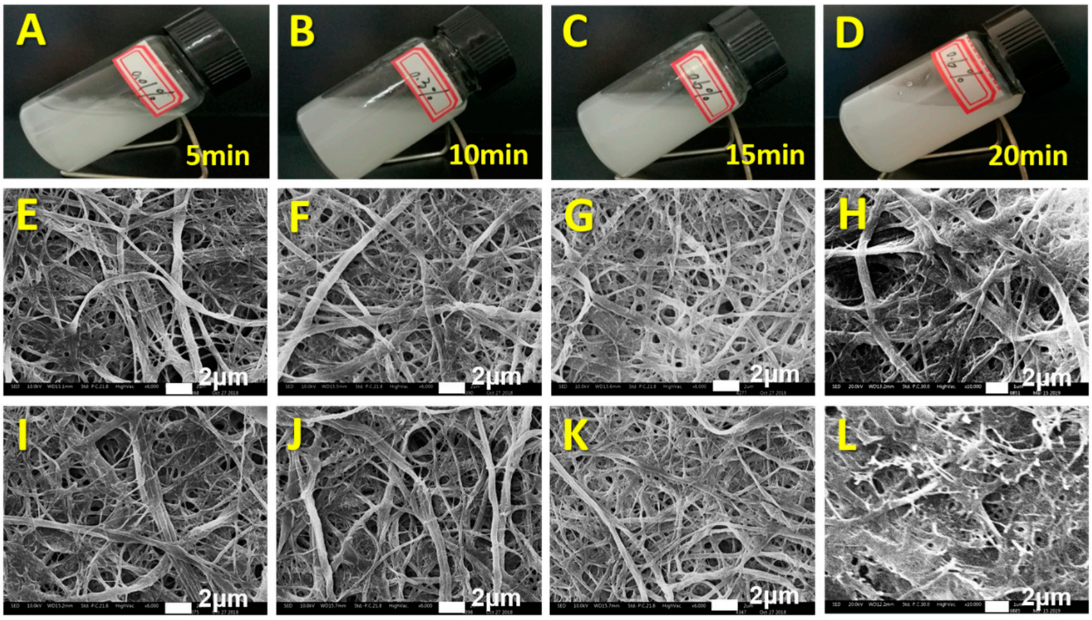

Figure S1 The SEM images of silk micro-nanofibers and SNF films. (A–D) represents the gross appearance of the SNFs obtained by different treatment times, E-H) Silk fibers treated for different times at 30 °C. I–L) Silk fibers treated for different times at 35 °C, scale bar = 2μm.
